# First-time endoscopic submucosal implantation of a wireless pacemaker device as a gastric submucosal electrical stimulator in a porcine model

**DOI:** 10.1055/a-2410-3010

**Published:** 2024-09-25

**Authors:** Antoine Debourdeau, Mathilde Sanavio, Veronique Vitton, Philippe Onana Ndong, Marc Barthet, Jean-Michel Gonzalez

**Affiliations:** 1 Hepatogastroenterology Department, Nimes University Hospital, CHU Nimes, Nimes, France; 2 Hepatogastroenterology Department, Montpellier University Hospital, CHU Montpellier, Montpellier, France; 3 Department of Gastroenterology, Hôpital Nord, AP-HM, Marseille, France; 4 Endoscopy Unit, Gastroenterology, Nice University Hospital, CHU Nice, Nice, France

Gastric electrical stimulation has been identified as a valuable intervention for relieving symptoms associated with gastroparesis, utilizing a surgically implanted device at the antro-fundic junction for high-frequency, low-energy impulse delivery 1 . Attempts have been made to implant stimulation devices in a minimally invasive manner, yet these were not sustained over time 2 3 .

The Micra device (Medtronic, Minneapolis, Minnesota, USA), a wireless miniature pacemaker originally developed for cardiac applications 4 , has been repurposed in this study to be used as a gastric stimulator. This device, introduced via a 23-French transfemoral catheter and measuring 2.6 × 0.7 cm ( Fig. 1 , Fig. 2 ), offers a novel approach to gastric electrical stimulation.

**Fig. 1:**
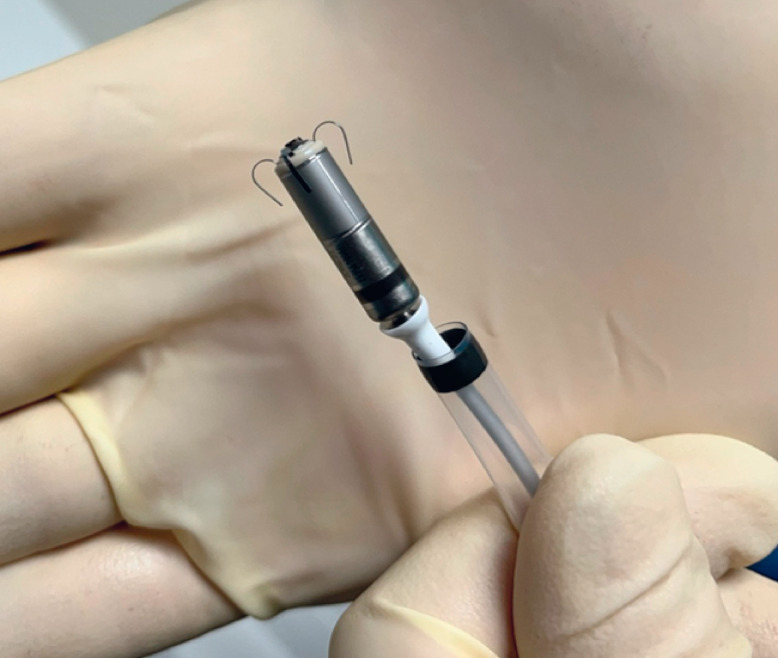
Micra device (2.6×0.7 cm).

**Fig. 2:**
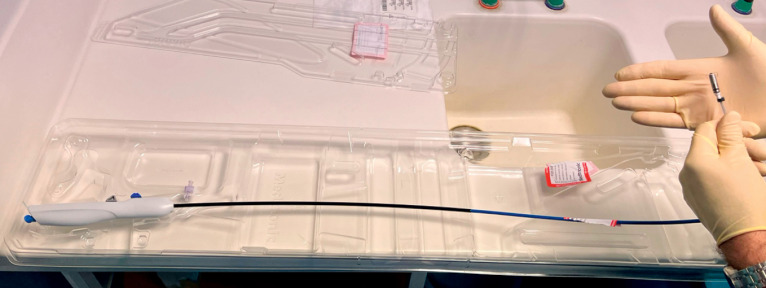
Micra device catheter.

In a procedural experiment conducted on a 35.5-kg female pig under general anesthesia, an implantation site was identified on the greater curvature at the antrum-fundus junction by analogy to what had been described for the placement of the Enterra device (Medtronic) 1 .

We performed a submucosal injection followed by a longitudinal incision to create a 50 × 60-mm pocket to house the device. After connecting the Micra implantation catheter to the endoscope in the 6 oʼclock position ( Fig. 3 ), we released the device into the pocket, then pushed it to the bottom using a rat tooth grasper. The longitudinal incision was then closed with the placement of endoclips ( Video 1 ).

**Fig. 3:**
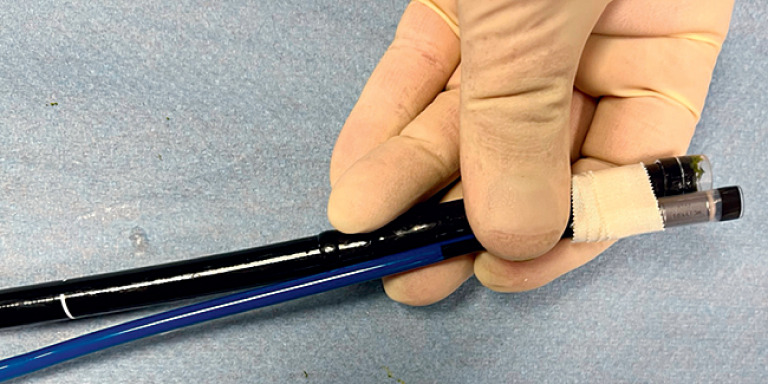
Connection of the Micra implantation catheter to the endoscope in the 6 oʼclock position.

Endoscopic submucosal implantation of a wireless pacemaker device as a gastric submucosal electrical stimulator in a porcine model.Video 1

The pig was monitored, and a subsequent intervention was carried out one month later to test the possibility of endoscopic removal of the device: we performed an “unroofing” technique with an incision of the mucosa on the superior pole of the submucosal protrusion caused by the device, and we were able to remove the Micra endoscopically. An autopsy of the pig confirmed the absence of peritonitis or injury to the gastric wall.

This approach demonstrates the technical feasibility of both implantation and removal of the Micra pacemaker in the stomach, paving the way for further studies on reproducibility and follow-up to envision its application in humans.

Endoscopy_UCTN_Code_CPL_1AN_2AD

## Notes

Endoscopy E-Videos https://eref.thieme.de/e-videos
*E-Videos* is an open access online section of the journal *Endoscopy* , reporting on interesting cases and new techniques in gastroenterological endoscopy. All papers include a high-quality video and are published with a Creative Commons CC-BY license. Endoscopy E-Videos qualify for HINARI discounts and waivers and eligibility is automatically checked during the submission process. We grant 100% waivers to articles whose corresponding authors are based in Group A countries and 50% waivers to those who are based in Group B countries as classified by Research4Life (see: https://www.research4life.org/access/eligibility/ ). This section has its own submission website at https://mc.manuscriptcentral.com/e-videos .
